# Voriconazole injection may induce delayed methotrexate excretion: a case report and experimental study

**DOI:** 10.1186/s40780-022-00240-3

**Published:** 2022-03-26

**Authors:** Daisuke Watahiki, Daisuke Saito, Naonori Nishida, Hiroyuki Tsuri, Keiko Nomura, Yuichi Adachi, Masato Taguchi

**Affiliations:** 1 Department of Pharmacy Practice and Sciences, School of Pharmacy and Pharmaceutical Sciences, University of Toyama, 2630 Sugitani, Toyama, 930-0194 Japan; 2 Department of Pediatrics, Faculty of Medicine, University of Toyama, Toyama, Japan

**Keywords:** High-dose regimen, Methotrexate, Sulfobutylether-β-cyclodextrine, Phenolsulfonphthalein, Voriconazole

## Abstract

**Background:**

We report a case of delayed excretion of methotrexate (MTX) in a pediatric patient on high-dose MTX therapy in response to a change in the concomitant dosage of voriconazole from oral to intravenous. As the intravenous, but not the oral formulation of voriconazole includes sulfobutylether-β-cyclodextrin (SBECD), which has an anionic residue, we hypothesized that SBECD inhibits the renal excretion of anionic compounds.

**Methods:**

We evaluated the inhibitory effects of SBECD on renal excretion of phenolsulfonphthalein (PSP), which is eliminated in urine via organic anion transport systems. PSP was administered intravenously to rats at 2.5 and 25 mg/kg with or without SBECD pretreatment (320 mg/kg).

**Results:**

The plasma concentration of PSP at the dosage of 2.5 mg/kg were comparable between control and SBECD groups. On the other hand, at 25 mg/kg the elimination of PSP was delayed. The clearance of PSP at the dosage of 25 mg/kg was 9.71 ± 1.65 and 4.13 ± 0.76 mL/min/kg in control and SBECD groups, respectively (*p* < 0.05). This suggested that SBECD partly inhibits the renal excretion of anionic drugs.

**Conclusion:**

The present case report discusses the delayed elimination of MTX in high dose therapy and possible mechanism involving SBECD as an excipient in concomitant drugs. It seems better to avoid choosing injection containing SBECD for patients undergoing HD-MTX treatment. Further studies are needed to confirm the inhibitory effects of SBECD on the renal excretion of MTX, especially in high-dose regimens.

## Introduction

Methotrexate (MTX) at 0.5 g/m^2^ and above, termed high-dose methotrexate (HD-MTX), is included in standard chemotherapeutic regimens for pediatric acute lymphoblastic leukemia (ALL) and its addition to treatment protocols has improved event-free survival [1, 2]. In HD–MTX treatment, delayed MTX elimination leads to fatal side effects such as mucositis, myelosuppression, renal failure, liver injury and neurotoxicity [1, 3]. Therefore, a regimen in Japan for pediatric patients with precursor B-cell ALL (JPLSG ALL-12) defines the therapeutic range of MTX in HD-MTX treatment as concentrations not exceeding 150 μM, 1.0 μM, 0.4 μM and 0.25 μM at 24 h, 42 h, 48 h and 66 h after the start of MTX administration, respectively [4]. In addition, supportive cares, such as hydration, urine alkalization, coadministration of diuretics, and monitoring of urine volume and pH, are needed to promote MTX excretion, and rescue with leucovorin is required to minimize the MTX toxicity [3–5]. If delayed excretion of MTX is observed, supportive care must be continued until the MTX concentration reaches 0.25 μM.

**Fig. 1:**
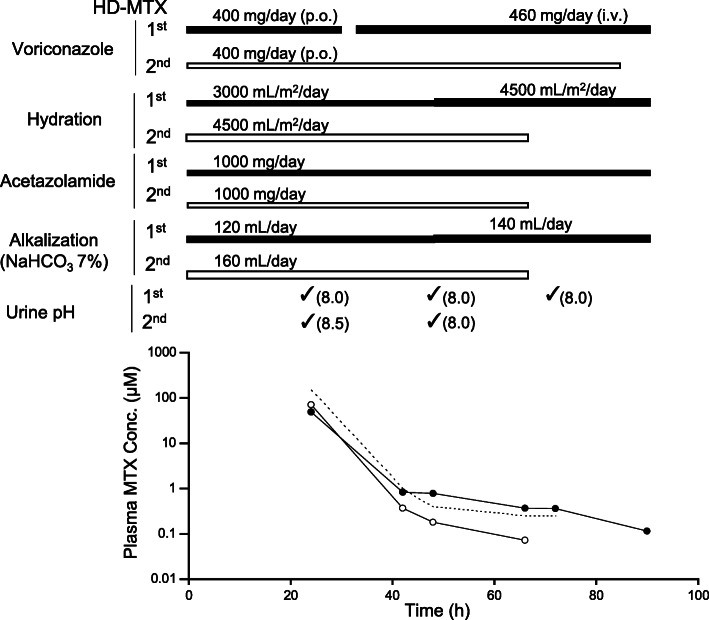
Plasma concentration of MTX and concomitant drugs in a patient who underwent HD-MTX treatment. Closed circle (●) and open circle (〇) represent MTX concentration at first and second rounds of HD-MTX treatment, respectively. The dotted line represents upper limit of MTX concentration on treatment protocol

We report a case of delayed excretion of MTX in the response to the coadministration of intravenous voriconazole in a pediatric patient with precursor B-cell ALL, who was undergoing the first course of HD-MTX treatment (Fig. 1). In the first course of HD-MTX, the MTX concentration was below the upper limit of the treatment protocol until 42 h after MTX infusion, but the MTX concentration at 48, 66 and 72 h exceeded the upper limit. Of note, the dosage form of voriconazole was changed from oral to intravenous at 32 h after MTX infusion (Fig. 1). Thus, we focused on evidence that intravenous, but not oral voriconazole includes sulfobutylether-β-cyclodextrin (SBECD), which improves the solubility of voriconazole [6, 7] (VFEND® I.V. contains 200 mg of voriconazole and 3200 mg of SBECD). SBECD is mainly excreted into urine and several reports suggested the accumulation of SBECD in patients with compromised renal function [6, 7]. In addition, because SBECD has anionic functional groups in its structure [6, 8], we hypothesized that SBECD affects the renal excretion of anionic compounds like MTX.

We conducted a preliminary verification study in rats at the dosage of 1 g/kg of MTX; however, only marginal effect of SBECD on MTX elimination was observed. Because experimental approach with higher doses of MTX (> 1 g/kg) was costly and difficult, we needed to consider alternative drugs. Thus we cited an article by Lin et al. [9]. That is, they used phenolsulfonphthalein (PSP) instead of MTX to investigate the interaction between MTX and another drug. The purpose of the present study was to report a clinical observation of delayed MTX excretion accompanied by voriconazole injection, and to demonstrate the inhibitory effects of SBECD on the renal excretion of PSP where renal excretion is saturated with high-dose.

## Materials and methods

### Clinical information about the patient

An 11-year-old male Japanese patient with precursor B-cell ALL was treated by HD-MTX at Toyama University Hospital. MTX was administered intravenously at 5 g/kg for 24 h. Maintenance fluid (Soldem 3A®; Terumo, Tokyo, Japan) was started to infuse at 3000 mL/m^2^/day from 24 h before infusion of MTX. As a combined medicine of HD-MTX treatment, 12 mg of MTX, 30 mg of cytarabine and 10 mg of predonisolone were intrathecally administrated at the same time. In addition, mercaptopurine (25 mg/m^2^) was taken orally before sleeping. No other drugs were administered under the HD-MTX therapy. The concentrations of MTX were monitored routinely. When MTX excretion was delayed, hydration, diuretic and urine alkalization were enhanced. The observational study period was from medical records over the period from November 2017 to December 2017. Information regarding HD-MTX treatment was extracted from the medical records, including demographics, plasma MTX concentrations, urine pH and volume, concomitant medications, and other medical diagnoses.

### Materials

PSP was purchased from Nacalai Tesque (Kyoto, Japan). SBECD was purchased from ChemScene, LCC (NJ, USA). All other chemicals and solvents were of the highest purity available.

### Animals and study protocols

Male Wistar rats (Sankyo Labo Service Corporation, Inc., Tokyo, Japan) aged 8 weeks old (200–250 g) were used. The rats were housed in a temperature- and humidity-controlled room with free access to standard rat chow and water before the experiment began.

Rats were anesthetized with medetomidine, midazolam and butorphanol. The femoral artery, jugular vein and urinary bladder were cannulated with a polyethylene tube (SP-31, Natsume Seisakusho, Tokyo, Japan) for blood sampling, intravenous administration and urine sampling, respectively. To exclude the influence of enterohepatic circulation, the bile duct was ligated. Body temperature was maintained with appropriate heating lamps. An 8% NaHCO_3_ solution was administered intravenously at 10 mg/kg between 30 and 25 min before MTX dosing. Then, mannitol was dissolved with saline (2%) and the solution was started to administer intravenously at 0.1 mL/min until the last blood sampling. Thirty minutes after NaHCO_3_ pretreatment, PSP was administered intravenously as a bolus in two different dosages, at 2.5 mg/kg and 25 mg/kg. Rats were pre-treated with intravenous saline (control) or intravenous saline containing SBECD 10 min before PSP dosing. The dose of SBECD was 320 mg/kg. Blood samples (220 μL) were collected just before PSP dosing, and at 1, 3, 10, 30, 60, 90 and 120 min after PSP dosing. Urine samples were collected before PSP dosing and at regular intervals of 30 min.

### PSP assay

The concentrations of PSP in plasma and urine samples were measured by the standard method with minor modification [10]. The samples were measured spectrophotometrically using a microplate reader (GENios™ F129004, Tecan, Austria) at 560 nm after appropriate dilution in 0.01 N NaOH. The quantitative limit of PSP was 3 μM.

### Pharmacokinetic analysis

The area under the blood concentration-time curve (AUC) of PSP after intravenous administration was calculated using the linear trapezoidal rule and extrapolated to infinity by dividing the last measureable concentration by the elimination rate constant (k_e_). The clearance (CL) was calculated from Dose/AUC. The renal excretion rate of PSP was obtained by dividing the amount of PSP excreted into urine by the dose of PSP.

### Statistical analysis

The significance of the differences between the two groups was evaluated by the Student’s t-test because all compared data were confirmed to be covariant. *P* < 0.05 was considered statistically significant. Data were expressed as the mean ± standard deviation.

## Results

Plasma MTX concentrations and concomitant drugs in the patient with delayed excretion during HD-MTX treatment are shown in Fig. 1. In the first course, renal and liver functions were normal and MTX concentrations were 49.2, 0.839, 0.788, 0.369, 0.364 and 0.115 μM at 24, 42, 48, 66, 72 and 90 h after MTX dosing, respectively (Fig. 1). Intravenous voriconazole was started from 32 h after MTX infusion, and MTX concentrations at 48 and 66 h exceeded the upper concentration limit in the protocol. Accordingly, hydration and alkalization were increased at 48 h and continued until the MTX concentration reached below 0.25 μM. In the second course, voriconazole was administered orally throughout the treatment. MTX concentrations were below the upper limit of treatment protocol, although hydration and alkalization were enhanced as compared with the first course and liver function seemed to be impaired slightly. That is, MTX concentrations were 70.8, 0.368, 0.181 and 0.073 μM at 24, 42, 48 and 66 h after MTX dosing, respectively (Fig. 1).

**Fig. 2:**
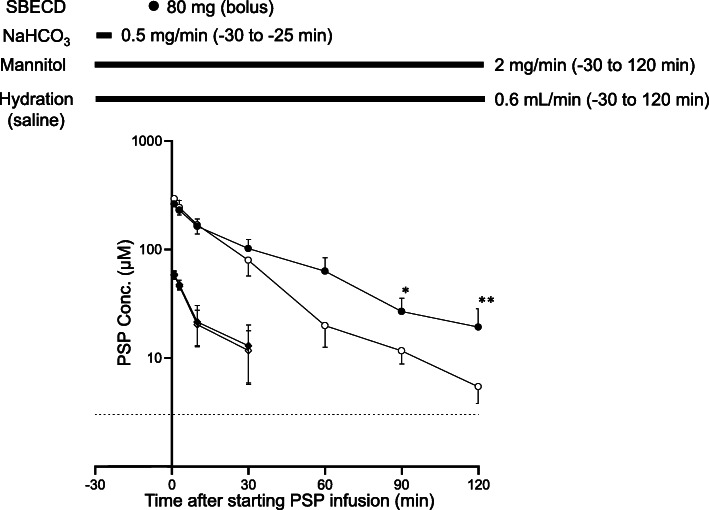
Experimental design for pharmacokinetic study and time course of PSP. Diamonds and circles represent PSP concentration at dose of 2.5 mg/kg and 25 mg/kg, respectively. Closed and open symbols correspond to SBECD and control groups, respectively. SBECD at 320 mg/kg (saline in control) was administered intravenously 10 min before the infusion of PSP. The dotted line represents quantitative limit of PSP (3 μM). *p < 0.05. ** *p* < 0.01

**Fig. 3:**
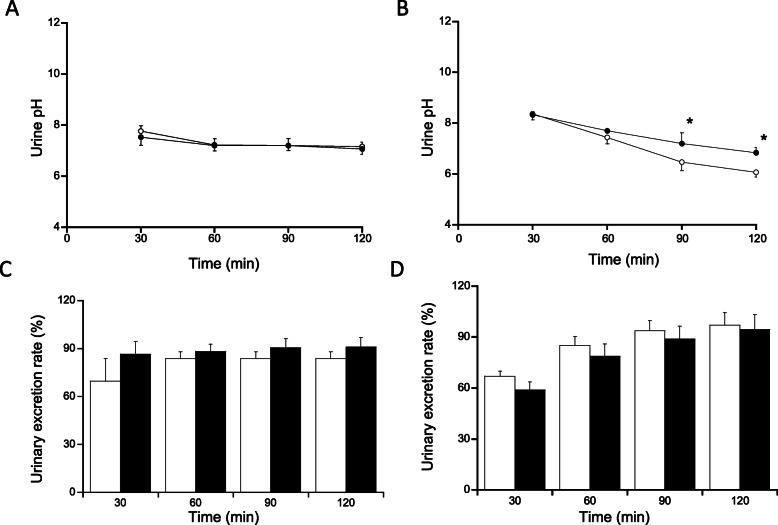
Urine pH and urinary excretion rate of PSP. PSP was administered intravenously at 2.5 mg/kg (**A, C**) and 25 mg/kg (**B, D**). Control group is represented by open circles and white columns. SBECD group is represented by closed circles and black columns. SBECD (saline in control) was administered intravenously at 320 mg/kg before the infusion of PSP. Each point and column represents the mean ± S.D. of three rats from two separate experiments. *p < 0.05

We next evaluated whether the delayed MTX excretion was induced by SBECD in rats. Instead of MTX, PSP was used as an anionic probe. PSP was administered at 2.5 and 25 mg/kg. The time course of the plasma concentration of PSP after intravenous administration in shown in Fig. 2. At 2.5 mg/kg, no effect of SBECD was observed, although we were unable to assess its pharmacokinetic parameters due to its quantitative limit. On the other hand, delayed elimination of PSP was observed in the SBECD group at 25 mg/kg (Fig. 2). Of note, the CL values significantly decreased and AUC values were significantly increased in SBECD group at 25 mg/kg (Table 1). Urine pH in rats treated with 25 mg/kg was higher in SBECD group than in control group, although urine pH was somewhat decreased in control rats (Fig. 3A, B). As the results, no significant difference in the urinary excretion rate of PSP between SBECD and control groups was observed (Fig. 3C, D).

**Table 1:** Pharmacokinetic parameters of PSP in rats

	2.5 mg/kg		25 mg/kg	
	control	SBECD	control	SBECD
ke (10^−3^ min^−1^)	ND	ND	10.2 ± 3.56	5.84 ± 1.63
CL (mL/min/kg)	ND	ND	9.71 ± 1.65	4.13 ± 0.76*
AUC (mmol·min/mL)	ND	ND	7.46 ± 1.32	10.9 ± 0.44*

PSP was administered intravenously at 2.5 mg/kg or 25 mg/kg. SBECD was intravenously administered at 320 mg/kg. Each value represents the mean ± S.D. (*n* = 3). *p < 0.05

ND: not determined due to quantitative limit

## Discussion

In the present study, we hypothesized that SBECD inhibits the renal excretion of anionic compounds. We performed preliminary experiments to investigate the effects of pretreatment with SBECD on MTX disposition, setting MTX dosage as 45 mg/kg (100 μmol/kg) or 1 g/kg (2.2 mmol/kg). At lower dose of MTX, the CL value was 22.9 ± 1.8 mL/min/kg and the AUC was 4.39 ± 0.26 mmol·min/mL in control group. In the SBECD group, the CL value was 27.1 ± 2.3 mL/min/kg and the AUC was 3.72 ± 0.33 mmol·min/mL. At 1 g/kg of MTX, the time course of plasma MTX concentration was only slightly elevated in the SBECD group. The CL value after 1 g/kg dose of MTX was 10.5 ± 2.0 mL/min/kg and the AUC was 217.8 ± 46.1 mmol·min/mL in control group. Similarly, the CL value was 8.45 ± 3.2 mL/min/kg and the AUC was 293.6 ± 89.7 mmol·min/mL in the SBECD group (data not shown). What is notable was that nonlinearity was observed in the MTX pharmacokinetics at the dosage of 45 mg/kg to 1 g/kg. However, we failed to demonstrate a conclusive effect of SBECD on the MTX elimination with high-dose experiment directly due to the cost disadvantages. That is, if we set the dosage of MTX 5 g/kg to a rat weighting 250 g, it will cost about 45,000 Japanese yen per one rat.

We employed PSP as a probe substrate of organic anion transporters (OATs) to evaluate the inhibitory effects of SBECD on the renal excretion of anionic drugs. Lin et al. [9] investigated the transporter-mediated interaction between indican and MTX based on the kinetic analysis of PSP disposition in rats. In their study, 5 mg/kg of PSP was administered intravenously with or without indoxyl sulfate, which is the metabolite of oral indican that is excreted into urine via OATs. The CL value was 3.1 ± 0.4 mL/min/kg and 1.0 ± 0.3 mL/min/kg in the absence and presence of indoxyl sulfate, respectively. They concluded that indoxyl sulfate can inhibit renal MTX excretion [9]. Based on these report, we set the dosage of PSP at 2.5 mg/kg and 25 mg/kg in the present study.

Itagaki et al. [11] demonstrated that PSP is cleared from peritubular blood by rat kidney organic anion transporters, particularly rat OAT1 (rOAT1) and rOAT3, and they evaluated the role of rOAT1 and rOAT3 in the renal uptake of PSP. In their study, the uptake of PSP in rats kidney slices was saturated at a high drug concentration. The Km value for the uptake of PSP by rOAT3, a high-affinity transporter, was 33.1 μM, whereas that for the uptake of PSP by rOAT1, a low-affinity transporter, was 1011 μM [11]. The maximum plasma PSP concentrations observed after 2.5 mg/kg and 25 mg/kg dose of PSP were approximately 50 μM and 300 μM, respectively (Fig. 2). Considering our results that the inhibitory effects of SBECD on PSP excretion was observed at 25 mg/kg but not at 2.5 mg/kg (Fig. 2, Table 1), the inhibitory effects of SBECD may occur under extreme conditions where renal excretion is at least partly saturated. In addition, Ginneken et al. [12] reported that when transport of an anionic drug in the brush border membrane of the tubular cell is much lower than that in the basolateral membrane, the drug accumulates in the tubular cell. This may explain the marginal inhibitory effects of SBECD on the urinary excretion of PSP (Fig. 3C and D).

The present study demonstrated the potential of SBECD to inhibit the renal excretion of anionic drugs in rats. To our knowledge, however, no drug-drug interactions involving SBECD has been reported, and the safety of SBECD was confirmed in several reports [6, 7, 13]. For example, Hoover et al. [7] demonstrated the safety of SBECD in patients with varying degrees of renal failure. After dosing with intravenous delafloxacin, which includes SBECD, the mean AUC of SBECD increased 1.3-, 2.2-, and 5.5-fold in subjects with mild, moderate, and severe renal impairment compared with normal patients, respectively. However, greater exposure to SBECD did not markedly increase treatment-related adverse effects [7]. Based on these results, it seems unlikely that SBECD caused renal impairment in our study. On the other hand, there is a case report about voriconazole-induced delayed MTX elimination with high-dose therapy. Bogaert et al. [14] reported a case with T-cell lymphoblastic lymphoma treated with MTX at 5 g/m^2^. The patient received secondary prophylaxis with voriconazole and underwent delayed MTX excretion (MTX level at 48 h was 4.067 μM) [14]. Our findings may explain one of the mechanisms in the case, although the information about dosage form and time schedule of voriconazole administration are missing in their work [14].

### Study limitation

In the HD-MTX treatment protocol, urine alkalization is conducted repetitively in order to maintain a urine pH over 7.0. In the present experiment, however, we were unable to adjust the urine pH constantly due to the time constraints of the experimental procedure. In addition, it was difficult to demonstrate an inhibitory effect of SBECD on renal MTX excretion in rats with sufficient dosing of the drug due to cost limitations. Finally, we should be careful to extrapolate our results to humans because there is species difference in expression of OATs and some species differences in substrate recognition and transport activity between rOAT1 and hOAT1 was reported [15].

## Conclusion

The present case report discusses the delayed elimination of MTX in high dose therapy and possible mechanisms involving SBECD as an excipient in concomitant drugs. It seems better to avoid choosing injection containing SBECD for patients undergoing HD-MTX treatment. Further studies are needed to confirm the inhibitory effects of SBECD on the renal excretion of MTX, especially in high-dose regimens.

## Data Availability

The datasets used and/or analyzed during the current study are available from the corresponding author on reasonable request.

## Abbreviations

MTX: Methotrexate
HD: High dose
ALL: Acute lymphoblastic leukemia
SBECD: Sulfobutylether-β-cyclodextrine
PSP: Phenolsulfonphthalein
AUC: Area under the blood concentration-time curve
CL: Clearance
OAT: Organic anion transporter
rOAT: Rat organic anion transporter
